# Canonical Wnt Signaling Activity in Early Stages of Chick Lung Development

**DOI:** 10.1371/journal.pone.0112388

**Published:** 2014-12-02

**Authors:** Rute Silva Moura, Eduarda Carvalho-Correia, Paulo daMota, Jorge Correia-Pinto

**Affiliations:** 1 Life and Health Sciences Research Institute (ICVS), School of Health Sciences, University of Minho, Braga, Portugal; 2 ICVS/3B’s - PT Government Associate Laboratory, Braga/Guimarães, Portugal; 3 Biology Department, School of Sciences, University of Minho, Braga, Portugal; 4 Department of Urology, Hospital de Braga, Braga, Portugal; 5 Department of Pediatric Surgery, Hospital de Braga, Braga, Portugal; Comprehensive Pneumology Center, Germany

## Abstract

Wnt signaling pathway is an essential player during vertebrate embryonic development which has been associated with several developmental processes such as gastrulation, body axis formation and morphogenesis of numerous organs, namely the lung. Wnt proteins act through specific transmembrane receptors, which activate intracellular pathways that regulate cellular processes such as cell proliferation, differentiation and death. Morphogenesis of the fetal lung depends on epithelial-mesenchymal interactions that are governed by several growth and transcription factors that regulate cell proliferation, fate, migration and differentiation. This process is controlled by different signaling pathways such as FGF, Shh and Wnt among others. Wnt signaling is recognized as a key molecular player in mammalian pulmonary development but little is known about its function in avian lung development. The present work characterizes, for the first time, the expression pattern of several Wnt signaling members, such as *wnt-1*, *wnt-2b*, *wnt-3a*, *wnt-5a*, *wnt-7b*, *wnt-8b*, *wnt-9a*, *lrp5*, *lrp6*, *sfrp1, dkk1, β-catenin* and *axin2* at early stages of chick lung development. In general, their expression is similar to their mammalian counterparts. By assessing protein expression levels of active/total β-catenin and phospho-LRP6/LRP6 it is revealed that canonical Wnt signaling is active in this embryonic tissue. *In vitro* inhibition studies were performed in order to evaluate the function of Wnt signaling pathway in lung branching. Lung explants treated with canonical Wnt signaling inhibitors (FH535 and PK115-584) presented an impairment of secondary branch formation after 48 h of culture along with a decrease in *axin2* expression levels. Branching analysis confirmed this inhibition. Wnt-FGF crosstalk assessment revealed that this interaction is preserved in the chick lung. This study demonstrates that Wnt signaling is crucial for precise chick lung branching and further supports the avian lung as a good model for branching studies since it recapitulates early mammalian pulmonary development.

## Introduction

All developmental processes are ultimately controlled by a cooperative action between several signal transduction pathways. Among the different pathways, Wnt signaling appears to be indispensable for orchestrating complex cell behavior that occurs during development [1], [2]. Wnt signaling is an intricate pathway that works in an autocrine or paracrine fashion. It involves cysteine rich ligand proteins (19 in human and mouse, and 18 encoded in the chick) [3] that activate intracellular pathways through the Frizzled (FZD) seven pass transmembrane receptors [4]. The activation of this pathway might also require co-receptors, such as the low-density lipoprotein receptor–related protein 5 (LRP5) and LRP6 [5], [6]. The complexity of this pathway is due to the fact that both the receptors and ligands involved in Wnt signal transduction belong to multi-gene families, allowing for a dazzling number of possible ligand-receptor interactions. The best known of these interactions, underlying the canonical signaling pathway [7], results in the activation of β-catenin/Tcf transcriptional complexes and leads to a variety of intracellular responses such as cell proliferation, differentiation, migration, adhesion, survival and death [8], [9]. In the absence of Wnt, cytoplasmic β-catenin associates with a destruction complex (that includes Axin among others), targeting the protein for degradation. When the pathway is activated β-catenin is translocated to the nucleus where it associates with transcription factors, activating transcription [1]. Moreover, canonical Wnt signaling is modulated by proteins such as secreted Frizzled-related proteins (sFRPs) and Dickkopf proteins (DKK) [1]. The non-canonical signaling is β-catenin independent and activates planar cell polarity (PCP) and Wnt/Ca^2+^ pathways [10].

Moreover, Wnt signaling has been shown to be involved in a variety of early embryonic events such as gastrulation [9], [11], somite patterning [12] and body axis formation [13], and also in the morphogenesis of numerous organs, namely lungs [14] and kidneys [15]. Furthermore, it has been implicated in limb [16], [17], nervous system [18], craniofacial [3] development and also in the cardiovascular system [19]. Hence, aberrant Wnt signaling pathway may result in serious malformations [1].

Lung development is a very complex process that depends on an elaborated crosstalk between the epithelium and the mesenchyme. Branching morphogenesis is tightly controlled by the interaction between several signaling pathways [20], namely FGF (Fibroblast Growth Factor), Notch, Shh (Sonic Hedgehog) and Wnt, that operate in both epithelial and mesenchymal compartments. Wnt-2b is essential for endoderm specification; embryos lacking *wnt-2b* expression exhibit complete lung agenesis since *Nkx2.1* expression is abrogated [21]. Several reports have shown the importance of canonical Wnt signaling early in branching morphogenesis, namely Wnt-7b in mesenchyme and epithelial proliferation [14], [22]. Actually, *wnt-7b* (−/−) mice die of respiratory failure because they display hypoplastic lungs and abnormal vascular development [14]. Non-canonical ligands such as Wnt-5a, appear to act in later stages [23]. A perturbation of this signaling pathway leads to an impairment of distal branching frequency since epithelial–mesenchymal interactions are compromised [11]. These findings are consistent with the lung phenotype observed in *wnt-5a* null and transgenic mice that present abnormal distal lung morphogenesis [23].

Furthermore, the complexity of lung branching regulation is also due to the fact that several signaling pathways interact with each other. In fact, a crosstalk between canonical Wnt and FGF signaling has been demonstrated; Shu *et al.* have shown that *fgfr2* (FGF10 cognate receptor) expression is dramatically reduced in lung airway epithelium in the absence of β-catenin, evidencing that FGF signaling is downstream of Wnt signaling in the mouse lung [24].

During chick development, the larynx and the trachea originate from the gut and, at day 3, two primordial lungs arise from this laryngotracheal groove by tracheal bifurcation [25]. These structures are built by a thin layer of endoderm and a thick covering of mesoderm. The two buds will then grow in a posterior direction forming the air passages and air-sacs, establishing a series of closed circular buds arising from the main airways branches. The avian lungs are composed by a looping anastomotic network of air and vascular surfaces, the parabronchi (the gas exchange unit). This complex architecture produces a remarkably efficient respiratory system [25]. As it occurs in the mammalian case [26], FGFs are important during chick lung branching [27] since FGF signaling inhibition leads to branching impairment; moreover, variation of FGF10 diffusion rate between ventral and dorsal regions seems to account for ventral cyst formation and dorsal branching morphogenesis [28]. Also, the developing respiratory tract presents region-specific mesenchymal expression of the *Hoxb* genes, among others [29], that govern differences between dorsal and ventral compartments of the lung. Avian and mammalian lungs are quite different in their adult state, mainly due to the high metabolic rate of birds, which requires a special arrangement and architecture of the respiratory system [25]. Despite these differences, the events underlying lung development at early stages seem to be the same [27]. However, while mammalian lung organogenesis is well studied [30], [31] there is still little knowledge about the molecular mechanisms of chick lung branching process.

In this report, we characterize for the first time the expression pattern of several Wnt ligands, such as *wnt-1*, *wnt-2b*, *wnt-3a*, *wnt-5a*, *wnt-7b*, *wnt-8b*, *wnt-9a*, *lrp5*, *lrp6*, *sfrp1, dkk1*, *β-catenin* and *axin2* by *in situ* hybridization. To assess whether the Wnt/β-catenin signaling pathway is active on the embryonic chick lung, we performed Western blot analysis of active and total β-catenin as well as LRP6 total and phosphorylated form. *In vitro* inhibition of canonical Wnt signaling pathway, by FH535 and PK115-584, and branching analysis was carried out in order to assess the role of Wnt signaling pathway in chick lung development. Moreover, Wnt-FGF signaling crosstalk was also evaluated.

## Materials and Methods

The work presented in this manuscript was performed in the chick model, at early stages of development, which doesn't need ethical approval from review board institution or ethics committee.

### Eggs and embryos

Fertilized chick (*Gallus gallus*) eggs, obtained from commercial sources, were incubated for 4–6 days in a 49% humidified atmosphere at 37°C. Embryonic chick lungs were carefully dissected under a dissection microscope (Olympus SZX16, Japan) and then classified in stage b1, b2, b3, taking into account the number of secondary buds formed, 1, 2 or 3, respectively [27].

### Western blot analysis

Pooled samples of embryonic chick lungs (3 pools per stage: 6 lungs per pool) and chick limb buds (HH24) were processed for western blot analysis. Proteins were obtained according to Kling *et al.* [32]. Five µg (for β-catenin studies) and 10 µg (for LRP6 studies) of protein were loaded onto 7.5% acrylamide minigels, electrophoresed at 100 V at room temperature and then transferred to nitrocellulose membranes (Hybond -C Extra, GE Healthcare Life Sciences, UK). Blots were probed with antibodies to Non-phospho (Active) β-Catenin (Ser33/37/Thr41) (1∶5000; #4270, Cell Signaling Technology Inc., USA), total β-Catenin (1∶30000; #NBP1-54467, NOVUS Biologicals, USA), LRP6 (1∶1000; #3395, Cell Signaling Technology Inc.), phospho-LRP6 (Ser1490) (1∶1000; #2568, Cell Signaling Technology Inc.) according to the manufacturer’s instructions. For loading control, blots were probed with β-tubulin (1∶200000; #ab6046, Abcam Inc., UK). Afterwards blots were incubated with a secondary horseradish peroxidase conjugate (Cell Signaling Technology Inc.), developed with Super Signal West Femto Substrate (Pierce Biotechnology, USA) and the chemiluminescent signal was captured using the Chemidoc XRS (Bio-Rad, USA). Three independent experiments per pool were performed. Quantitative analysis was performed with Quantity One 4.6.5 1-D Analysis Software (Bio-Rad). All quantitative data are presented as mean ± SEM. Statistical analysis was performed, using SigmaStat 3.5 (Systat Software Inc., USA), by One Way ANOVA and Holm-Sidak test was used for post-test analysis. Statistical significance was set at p<0.05.

### Lung Explant Culture

After dissection in DPBS (Lonza, Switzerland) lungs were transferred to Nucleopore membranes with an 8 µm pore size (Whatman, USA) and incubated in a 24-well culture plates (Orange Scientific, Belgium). The membranes were presoaked in 400 µL of Medium 199 (Sigma, USA) for 1 h before the explants were placed on them. Floating cultures of chick lung explants were performed as previously described [27]. The branching morphogenesis was monitored daily by photographing the explants. At d0 (D0∶0 h) and d2 (D2∶48 h) of culture, the total number of peripheral airway buds (branching) was determined. The results of branching were expressed as D2/D0 ratio. All quantitative morphometric data are presented as mean ± SEM. Statistical analysis was performed, using SigmaStat 3.5 (Systat Software Inc., USA), by One Way ANOVA on Ranks and Dunn’s test was used for post-test analysis. Statistical significance was set at p<0.001.

In order to inhibit Wnt signaling pathway, lung explants (stage b1 to b3) were cultured with FH535 (Sigma), a β-Catenin/Tcf inhibitor. FH535, dissolved in DMSO, was added to the medium to achieve a final concentration of 20, 30 and 40 µM (n≧15, per stage) and 0.1% DMSO. Lung explants (stage b2) were cultured with PK115-584 (Calbiochem, UK), that disrupts β-catenin/Tcf4 complex. PK115-584 (also known as calphostin C), dissolved in DMSO, was added to the medium to achieve a final concentration of 1 and 2.5 µM (n≧14) and 0.59% DMSO. Control explants consisted of medium containing DMSO at a final concentration of 1 µL/mL for FH535, and 5.9 µL/mL for PK115-584 (n≧15, per stage). After culture, lung explants were fixed overnight at 4°C and processed for *in situ* hybridization or TUNEL assay.

### *In situ* hybridization

Whole mount *in situ* hybridization (n = 15 per stage and probe) was performed as previously described [33]. Explants were processed simultaneously, developed for the same amount of time and photographed with an Olympus U-LH100HG camera coupled to Olympus SZX16 stereomicroscope.

### RNA Probes

Antisense digoxigenin-labeled RNA probes were produced as previously described: *wnt-1*, *wnt-3a* and *wnt-8b* [18], *wnt-5a* and *wnt-7b* [34], [35], *β-catenin* [36], *axin2* [37], *srfp1* [38], *dkk1* [3], *frfr2b* [39]. *lrp5*, *lrp6*, *fgf10* and *spry2* probes were generated by RT-PCR from stage 24 whole chick embryo RNA using oligonucleotides. Lrp5: forward 5′-ctggaggtacaaagctggagt-3′ and reverse 5′-aaaggggagtgtgaaagtagggct. Lrp6: forward 5′-taatcgtgggtggcctcgaa and reverse 5′-aattaaccctcactaaaggaattccacatgggtttgtagca. Fgf10: forward 5′-ttataaaagcttgcggccgcagaatataccaggttttacccatccagtatg-3′ and reverse 5′-gctctagaaattaaccctcactaaaggttgtggctccccttccattc-3′. Spry2: Forward 5′-ttataaaagcttgcggccgcagaatatctgctccaacgatgatgaggac-3′ and reverse 5′-gctctagaaattaaccctcactaaaggaggggtgacacttgtaagatgcc-3′. Chick probes for *wnt-2b* and *wnt-9a* were kindly provided by Dr. Rodriguez-León (IGC, Portugal).

### TUNEL assay

Apoptosis in chick lung explants was analyzed using the Cell Death Detection Kit (Roche Applied Sciences, Germany). Lung explants were fixed overnight in 4% paraformaldehyde (PFA) in PBS and TUNEL assay was performed as previously described [27].

### Cross-Section Preparation

Hybridized chick lungs were fixated in paraformaldehyde 4%, embedded in 2-hydroxyethyl methacrylate (Heraeus Kulzer, Germany) and processed for sectioning at 25 µm using a rotary microtome (Leica RM 2155, Germany). Lung sections were photographed with an Olympus DP70 camera coupled to an Olympus BX61 microscope.

## Results and Discussion

Wnt signaling pathway is a major player during vertebrate’s embryonic development that has been associated with several developmental processes. Wnt signaling has been classically divided in canonical and non-canonical pathway, and the majority of Wnt ligands have the ability to activate both pathways [40]. Canonical Wnt signaling, by far the most studied one, signals through FZD receptors and LRP co-receptors and depends on β-catenin stabilization for its activation (reviewed in [1]). Non-canonical pathway also comprises the activation of FZD receptors, but instead of cascading through β-catenin, it activates other intracellular pathways like planar cell polarity and the Wnt/Ca^2+^. In the PCP pathway, Wnt activates Jun-N-terminal Kinase (JNK) and acts on cytoskeletal organization and cell polarization. In the Wnt/Ca^2+^ pathway, Wnt activation leads to the release of intracellular calcium, possibly through mediators like G-proteins, and affects cell adhesion [10]. It has been recently shown that PCP pathway is required for normal lung development since loss-of-function mouse mutants affecting core proteins of this pathway display smaller lungs with a reduced number of epithelial branches [41], [42]. Nonetheless, to the best of our knowledge, there are only few reports dedicated to the study of non-canonical pathways in lung development.

On the other hand, canonical Wnt signaling has been widely studied, and as stated previously, it is known to play an important role in some aspects of lung development such as: specification of the foregut [21], mid-to-late gestational airway and vascular patterning [43], and lung mesenchymal growth/differentiation and vascular development [14]. So far, the expression pattern of this signaling pathway has not been characterized in chick lung development.

In this study we have examined transcript location, by *in situ* hybridization, of some members of the Wnt signaling pathway at early stages of chick lung development such as *wnt-2b*, *wnt-5a*, *wnt-7b*, *wnt-9a*, that are known to be involved/present in mammalian lung development, and other canonical ligands such as *wnt-1*, *wnt-3a* and *wnt-8b*. Canonical Wnt signaling pathway relies on several components such as co-receptors, as *lrp5* and *lrp6*, and regulators as for instance *srfp1* and *dkk1*, which have also been characterized; it has several target genes, including members of the protein activation complex, such as *axin2* that was likewise analyzed. Additionally, the expression pattern of *β-catenin* (which is not a downstream target of Wnt signaling pathway) was also examined. Representative examples of hybridized lungs from different stages of development were sectioned for histology. Moreover, Wnt inhibition studies were performed in order to assess Wnt’s functional role in early branching morphogenesis. Additionally, protein levels of β-catenin (total and non-phosphorylated) and LRP6 (total and phosphorylated) were evaluated. Wnt-FGF crosstalk was also assessed.

### Expression pattern of *wnt-1*, *wnt-2b*, *wnt-3a*, *wnt-5a*, *wnt-7b, wnt-8b* and *wnt-9a* during chick lung development

**Figure 1:**
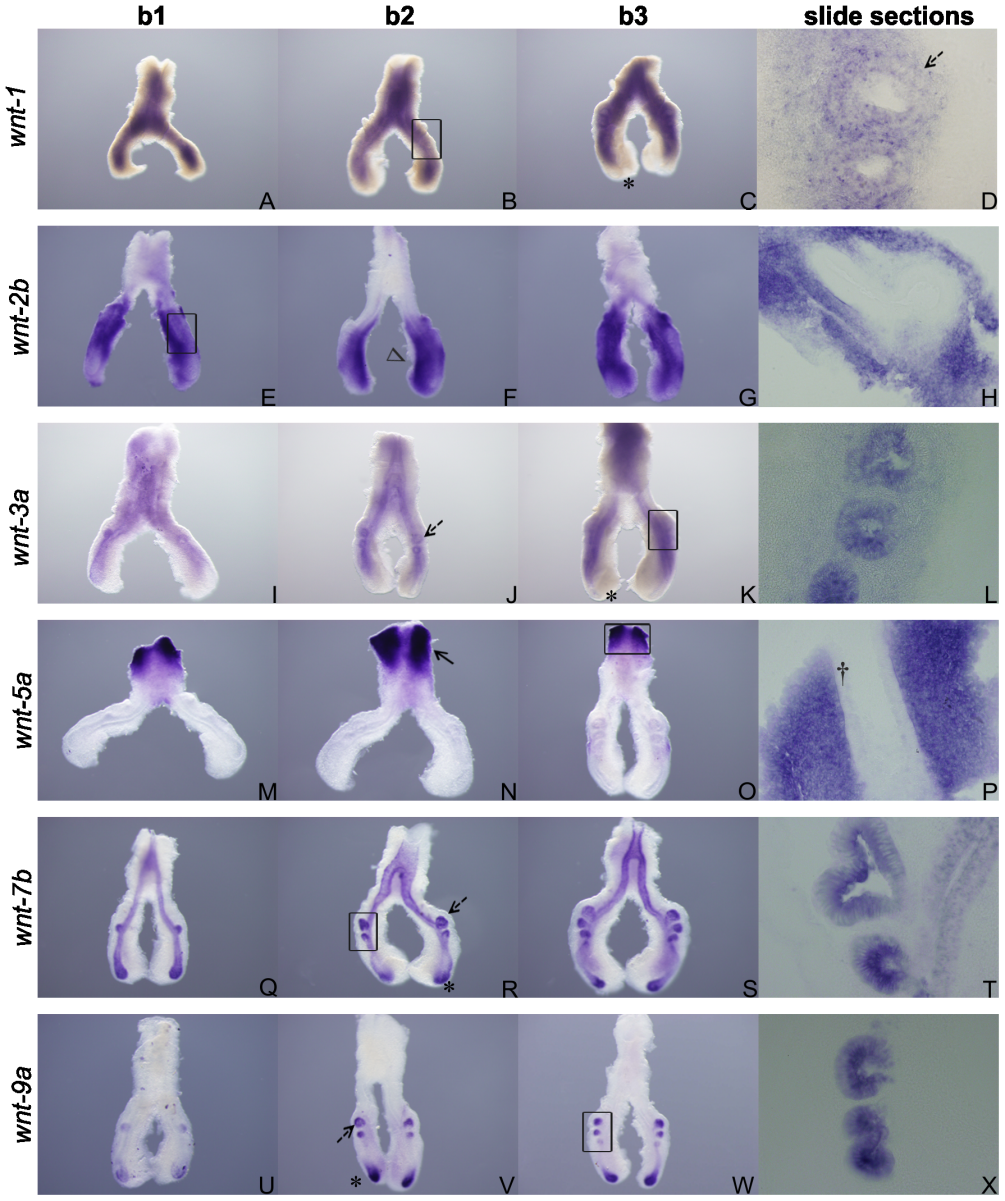
Wnt ligands expression pattern in early stages of chick lung development. Representative examples of *in situ* hybridization of *wnt-1* (A–D), *wnt-2b* (E–H), *wnt-3a* (I–L), *wnt-5a* (M–P), *wnt-7b* (Q–T), *wnt-9a* (U–X) of stage b1, b2 and b3; n = 15 per stage. Open arrowhead – medial mesenchyme. Black arrow – proximal mesenchyme. Dagger – proximal epithelium. Asterisk – epithelial tip of the main bronchus. Dashed black arrow – secondary buds epithelium. Magnification: whole mount, 5×; slide sections, 20×. The black rectangle in images B, E, K, O, R and W indicate the region shown in corresponding slide section.

*wnt-1* expression in the chick embryonic lung appears to be present mainly in the mesenchyme that surrounds the epithelial compartment (Figure 1A–C). Conversely, it seems to be absent from the epithelial tip of the main bronchus (Figure 1C, asterisk). This pattern is consistent in the three stages studied. Slide sectioning revealed that expression is not restricted to the mesenchymal compartment and that *wnt-1* is also expressed in the epithelium (Figure 1D, dashed black arrow) although it is absent in the most distal region. There are no studies regarding *wnt-1* expression/function during mammalian fetal lung development. In human adult lung Wnt-1 is mainly expressed in bronchial and alveolar epithelium and also in vascular smooth muscle cells [44]. There are evidences that anomalous activation of Wnt-1 signaling is associated with a variety of human malignancies including lung cancer [45], [46] and other respiratory diseases such as idiopathic pulmonary fibrosis [47]. Wnt-1 oncogenic potential is due to the fact that it inhibits apoptosis and promotes cell survival of cancer cells [48]. It has also been described that *wnt-1* is required for proper development of the entire mid−/hindbrain region [49] and that controls proliferation of specific progenitor cell populations [50]. Probably, in the fetal lung, *wnt-1* might be also implicated in the proliferation of specific cell types.

*wnt-2b* is present mainly in the mesenchyme surrounding the main bronchi particularly in the medial region (Figure 1F, open arrowhead), and almost absent from the most proximal area, the trachea, in the three stages studied (Figure 1E–H). Histological sectioning of hybridized lungs confirmed the mesenchymal expression, higher in the medial area (Figure 1H). Moreover, it also showed that there is no expression in the pulmonary epithelium. These results are consistent with those of its homologue in mice where both *wnt-2* and *wnt-2b* are expressed in the mesenchyme, and that are known to have an essential role in lung specification of the foregut [21].

*wnt-3a* appears to be expressed in the epithelium of the secondary bronchi (Figure 1J, dashed black arrow) and in the main bronchus, except in distal tip (Figure 1K, asterisk). There are no differences between the three stages studied. Slide sectioning revealed that the expression is lacking from the mesenchymal compartment; it is expressed in the epithelial compartment (Figure 1L), however in the distal tip of the main and secondary bronchus *wnt-3a* mRNA is absent (as it occurs with *wnt-1*). This expression pattern is in agreement with a former study that showed that *wnt-3a* is expressed in the epithelial compartment of E7 chick lungs. Latter in development, by E12, it is strongly expressed in the interstitial vasculature and the distal epithelium but not in the mesenchyme [51]. The same authors demonstrated that Wnt-3a canonical signaling is associated with early-proximal airway development [51].

*wnt-5a* expression in the chick lung is confined to the most posterior area of the respiratory tract, specifically the trachea (Figure 1N, black arrow), and no expression is observed in the main bronchi (Figure 1M–P). Slide sectioning confirmed that *wnt-5a* expression is only present in the mesenchymal compartment (Figure 1P). No differences were observed in the three stages studied. In embryonic mouse lungs (E12), this gene is greatly expressed in distal lung epithelium and surrounding mesenchyme; however the highest level of *wnt-5a* expression is localized to the area of the pharynx [23], which is consistent with the expression pattern in the chick lung. De Langhe *et al.* [52] also described low levels of expression in the mesenchyme and epithelium, and high levels around the pharynx and proximal trachea. A previous study carried out in chick embryos showed that by E7 *wnt-5a* appears to have no expression in the lung, but is already expressed in the esophagus. After embryonic day 11, *wnt-5a* expression is detected in both lung epithelium and adjacent mesenchyme, suffering a declination in the mesenchyme until hatching, becoming exclusively epithelial [51].

While canonical Wnt pathway is known to regulate lung development early in branching morphogenesis, non-canonical pathway activators such as Wnt-5a seem to have an important role in mid-to-late gestational stages of airway and vascular patterning (during alveolarization) [23], possibly through regulation of other major pathways (FGF and Shh). In fact, Wnt-5a mis/overexpression leads to abnormal chick lung phenotype from E10 onwards [51]. On the other hand, *wnt-5a* (−/−) lungs present the correct number of lobes, nevertheless a truncation of the trachea occurs and abnormalities in distal lung architecture are detected without affecting pulmonary cellular differentiation [23]. Chick *wnt-5a* expression is confined to the tracheal region which might indicate a possible role of this gene in the morphogenesis of the proximal area of the lung at early stages of development [51].

*wnt-7b* is clearly expressed throughout all pulmonary epithelium not only in the main bronchus, particularly in the tip (Figure 1R, asterisk), but also in secondary bronchi (Figure 1R, dashed black arrow), in the three stages studied. *wnt-7b* transcript seems to be absent from the lung mesenchyme (Figure 1Q–T). Histological sectioning of hybridized lungs confirmed this expression pattern (Figure 1T). In mouse embryonic lung *wnt-7b* is also expressed in the developing airway epithelium [52]. It has been shown that epithelial Wnt-7b signaling is required for proper lung epithelial and mesenchymal growth/differentiation and vascular development [22]. Actually, *wnt-7b* (−/−) mice die perinatally as a result of lung hypoplasia which is due to a decrease in early mesenchymal proliferation, from E12.5 onwards, and severe defects in the smooth muscle of the major pulmonary vessels leading to an extensive hemorrhage at birth [14]; this event is a clear example of how inductive interactions between mesenchyme and epithelium are crucial to lung patterning. *wnt-7b* chick lung expression pattern is in agreement with its mammalian counterpart which might indicate a similar regulatory function for this gene in the chick.

*wnt-8b* is not expressed in embryonic chick lung in the three stages studied (data not shown). This result indicates that at early stages of chick lung branching, this ligand doesn’t intervene. So far, this gene had only been reported in the developing chick brain [18], [53]. To the best of our knowledge this ligand has not been described in mammalian lung development.

*wnt-9a* is detected only in the growing tips of the lung, namely in the tip of the main bronchi (Figure 1V, asterisk) and also in the secondary buds (Figure 1V, dashed black arrow); no expression is observed in the remaining epithelial compartment and the mesenchyme (Figure 1U–X). This expression pattern was confirmed in lung sections (Figure 1×). It had already been described in *Xenopus* the presence of this gene in the lung primordia and in lung buds but without further description [54]. *wnt-9a* expression pattern was already described in mouse [55] and chick limb development [56], and in chick hepatic [57] and eye development [58]. However, this is the first time that it is characterized, in detail, in early stages of lung development. The clear expression pattern of *wnt-9a* in the most distal areas of the lung, namely the peripheral airways and the tips of the main bronchi, suggests a role in lung growth, as occurs with *wnt-7b* that, despite having a clear epithelial location, is also important for mesenchymal proliferation.

**Figure 2:**
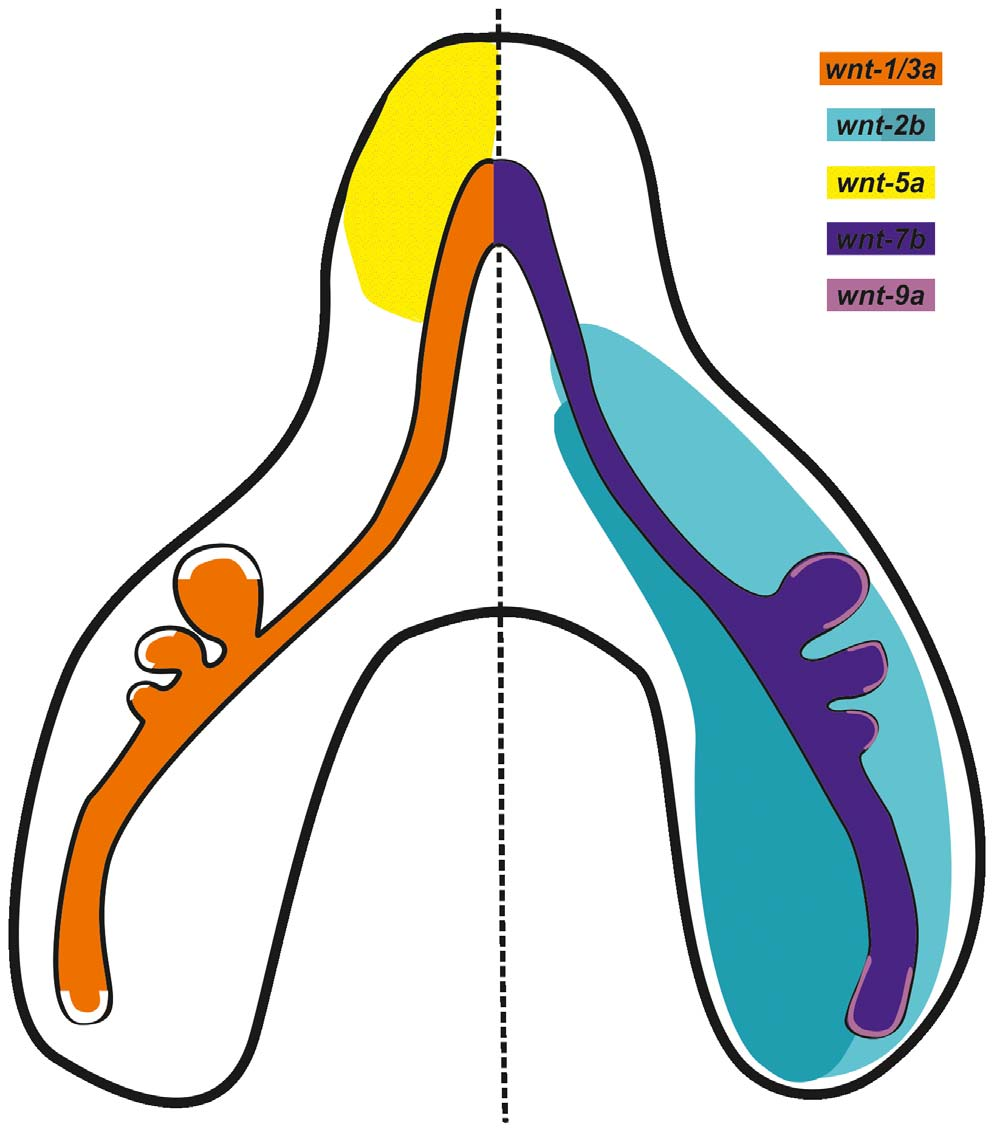
Schematic diagram of Wnt ligands expression pattern in a b3 stage lung. For simplicity, image was divided in half in order to avoid color overlay. Orange: *wnt-1* and *wnt-3a* expression; turquoise: *wnt-2b* expression; yellow: *wnt-5*a expression; dark blue: *wnt-7b* expression; purple: *wnt-9a* expression. Dual colors highlight different levels of expression.

A summary diagram of the Wnt ligands expression patterns is represented in Figure 2.

### Expression pattern of *lrp5*, *lrp6*, *sfrp1*, *dkk1, β-catenin* and *axin2* during chick lung development

**Figure 3:**
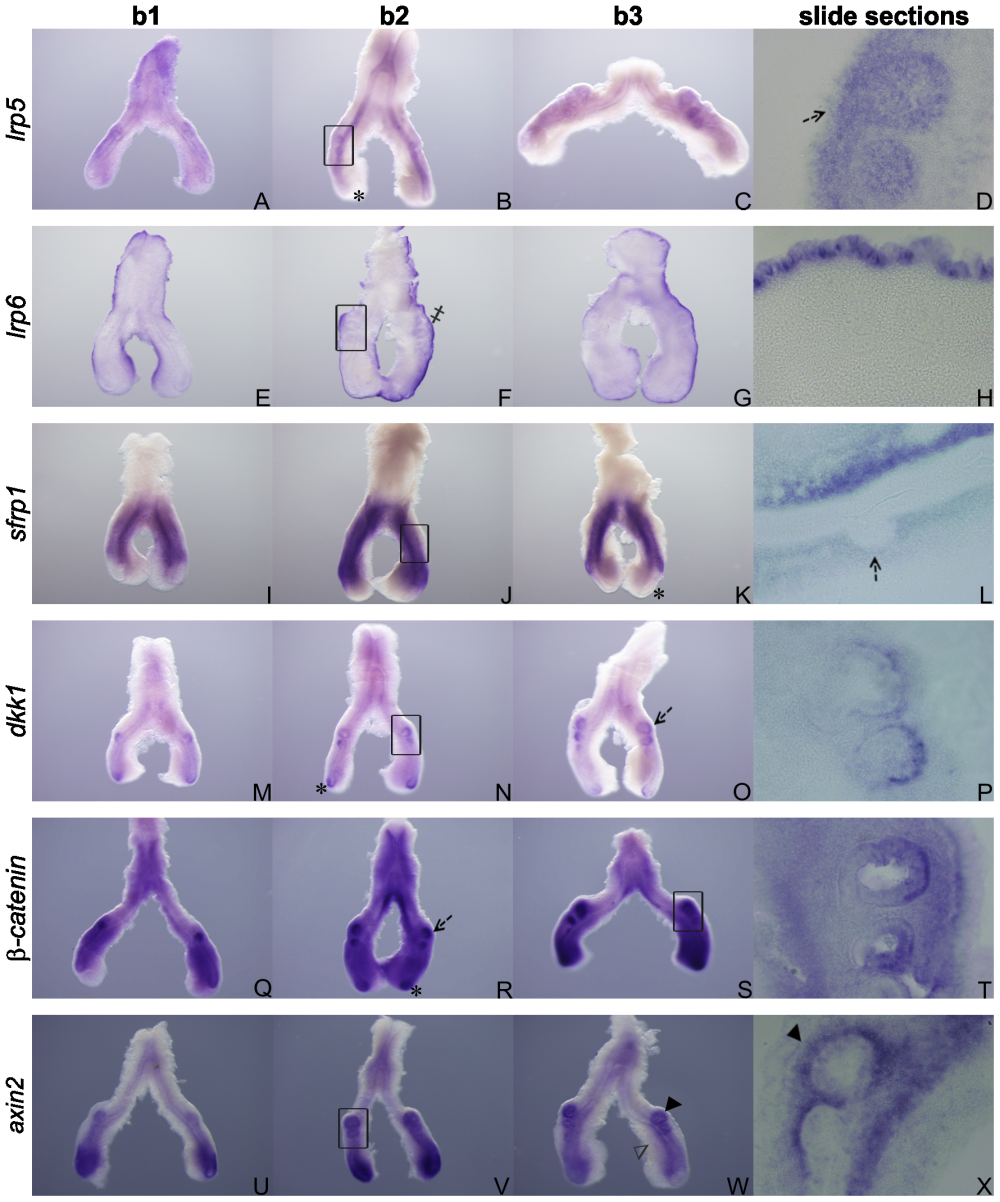
Wnt signaling members’ expression pattern in early stages of chick lung development. Representative examples of *in situ* hybridization of *lrp5* (A–D), *lrp6* (E–H), s*frp1* (I–L), *dkk1* (M–P), *β-catenin* (Q–T), *axin2* (U–X) of stage b1, b2 and b3; n = 15 per stage. Asterisk – epithelial tip of the main bronchus. Dark arrowhead – periepithelial mesenchyme of secondary buds. Double dagger – mesothelium. Dashed black arrow – secondary buds epithelium. Open arrowhead – medial mesenchyme. Magnification: whole mount, 5×; slide sections, 20×. The black rectangle in images B, F, J, N, S and V indicate the region shown in corresponding slide section.

*lrp5* mRNA is located in the epithelial compartment except in the distal tip of the main bronchus (Figure 3A–C), in the three stages studied, and slide sectioning confirmed this expression pattern (Figure 3D). In the murine lung *lrp5* is present in distal lung airway epithelium as early as E12.5, decreasing after E14.5 [59]. The same authors demonstrated that cell-specific activation of canonical Wnt signaling by Wnt-7b requires an interaction with LRP5 and with Fzd1 and 10. Taking into account that *wnt-7b* as well as *lrp5* expression patterns are similar to their mammalian counterparts it is plausible to think that the interaction between Wnt-7b and LRP5, which leads to the activation of canonical Wnt signaling, might be conserved in the avian lung.

*lrp6* is expressed in the outermost region of the lung, the mesothelium (Figure 3E–H, double dagger). Sections of hybridized lungs revealed that *lrp6* transcript is exclusively located in the cells that outline the embryonic lung (figure 3H). In the mammalian lung it has been suggested that the pleura regulates proliferation/differentiation of the mesenchymal compartment [60]. In fact, the mesothelial cells produce several signaling molecules, such as FGF9 [61] and Retinoic Acid [62], that are known mediators of these processes. Considering the intricate pathway crosstalk that occurs during development, it is possible that in the chick lung Wnt signaling contributes to this mechanism. This expression pattern is absolutely different from the distal airway epithelial expression described for mammalian lung [59]. In the avian lung, LRP5 and LRP6 are likely to play distinct roles due to their rather different expression patterns.

*sfrp1* expression is evident in the mesenchyme of the main bronchus (Figure 3I–K). On its turn it is clear that distal lung epithelium doesn’t express this gene (Figure 3K, asterisk), for all stages studied. Sectioning of the hybridized lungs revealed that *sfrp1* is clearly not expressed in the epithelium of secondary buds (Figure 3L, dashed black arrow). In the developing mouse lung *sfrp1* mRNA is found, from E10.5 to E14.5, in the mesenchyme adjacent to the epithelium [63] which is in accordance with the results obtained for the chick lung. In mice, loss of *sfrp1* disrupts proper alveolar formation since it alters the mesenchymal component surrounding the forming ductal units during lung development [64]. It is possible that, also in the chick lung, *sfrp1* might be important in the formation of distal structures.

*dkk1* is only detected at the growing ends of the epithelium specifically in the tip of main bronchus (Figure 3N, asterisk) and in the secondary bronchi (Figure 3O, dashed black arrow), in the three stages studied. Lung sections confirmed this restricted expression pattern (Figure 3P). These results are consistent with the ones described for the mouse lung in which *dkk1* is expressed, from E13.5 on, in the distal epithelium [52]. Overexpression of Dkk1 in lung epithelium resulted in the disruption of proximal–distal patterning revealing a role for canonical Wnt signaling in lung morphogenesis [52]. Considering the similar expression pattern in the chick lung, this mechanism is probably conserved in avian lung organogenesis.

*β-catenin* is expressed throughout all the mesenchyme of the developing chick lung; additionally it is highly expressed in the region adjacent to the secondary buds and to the tip of the main bronchus (Figure 3Q–T). No differences were observed in *β-catenin* expression pattern in the three stages studied. Lung sections confirmed this ubiquitous expression and showed an epithelial expression mainly in the tip of secondary buds and main bronchus (Figure 3R, dashed black arrow and asterisk, respectively). *β-catenin* is one of the most studied members of Wnt signaling pathway since it is responsible for the activation of transcription factors like Tcf/Lef, in the canonical Wnt pathway. In mouse, *β-catenin* is detected in epithelial cells lining the more peripheral lung tubules, and is not frequently observed in larger conducting airways. Additionally, it was shown that β-catenin signaling is required for the formation of the distal, but not the proximal, airways since it’s required for the determination of cell fate in the lung [43]. In human lung it is highly expressed in the peripheral epithelium [43]. Chick lung expression pattern is somehow consistent with the expression in mammalians, which might point to β-catenin as an important mediator in vertebrate lung development.

*axin2* mRNA is detected in the pulmonary peri-epithelial mesenchyme of the chick respiratory tract (Figure 3W, open arrowhead), in all stages studied; its expression is more intense in the peri-epithelial mesenchyme surrounding the secondary buds (Figure 3W, dark arrowhead) and the tip of the main bronchus. *axin2* transcript is absent from the epithelial compartment. Histological sectioning of hybridized lungs confirmed this expression pattern (Figure 3X). *axin2*, a direct readout of canonical Wnt signaling pathway, has been largely characterized during embryonic development [65]. In human fetal lung *axin2* is expressed mainly in the peripheral epithelium but also at low levels in the sub-adjacent mesenchyme [65]. The expression is observed in different compartments in these two models, but the localization is maintained: adjacent to the growing ends of the lung.

The expression patterns described in this study strongly suggest that in the chick lung, canonical Wnt signaling is involved in epithelium-mesenchyme interactions required for proper pulmonary branching since ligands, regulators and co-receptors of this pathway are specifically expressed in this tissue.

### Wnt signaling activity in the embryonic chick lung

**Figure 4:**
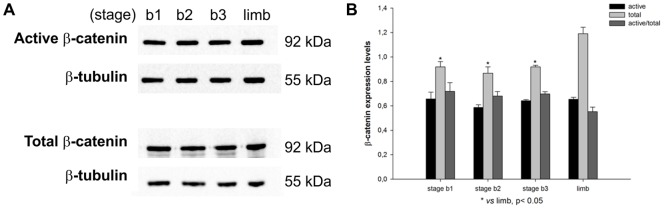
Activity of Wnt/β-catenin pathway in the embryonic chick lung. (A) Western blot analysis of active and total β-catenin in stage b1, b2 and b3 lungs, and stage 24 limb (as positive control). Control loading was performed using β-tubulin (55 kDa). Total and active β-catenin correspond to 92 kDa. (B) Semi-quantitative analysis for active and total β-catenin. Results are presented as arbitrary units normalized for β-tubulin. p<0.05: * *vs* limb.

In order to prove that canonical Wnt/β-catenin signaling is active in the developing chick lung, Western blot analysis was performed. The canonical Wnt pathway strictly controls the levels of cytoplasmic β-catenin. If β-catenin is phosphorylated in specific serine and threonine residues (Ser33/37/Thr41) it is targeted for ubiquitination and proteasomal degradation. Upon ligand binding to specific surface receptors, accumulation of non-phosphorylated (active) form of β-catenin in the cytoplasm occurs; then non-phosphorylated protein is translocated into the nucleus and regulates the expression of target genes such as *axin2* [66]. Pooled samples of embryonic lungs (stage b1, b2 and b3) were used to assess protein expression levels of non-phosphorylated (active) and total β-catenin (Figure 4). As a positive control, pooled samples of embryonic chick limbs (HH24) were included. Wnt signaling is active during limb development and it has been shown to have important functions during limb bud initiation, limb outgrowth, early limb patterning and later limb morphogenesis events [16].

As it can be observed from Figure 4A, the active form of β-catenin is present in lungs from the three stages studied demonstrating that Wnt signaling is active in this embryonic tissue. Semi-quantitative analysis showed total β-catenin is less expressed in lung samples that in limb. On the other hand, there are no differences in active β-catenin expression levels between the three lung stages and limb; the ratio between active/total β-catenin suggests that the degree of pathway activation is the same in both tissues (Figure 4B). Considering that limb is a good example of Wnt signaling activation, we can conclude that Wnt signaling is active in early stages of chick lung development and that there is no spatial-temporal regulation, at least in these stages.

Taking into account that β-catenin can also be activated by factors other than Wnt ligands [67] and considering that LRPs co-receptors are indispensable for canonical Wnt signaling, the levels of phospho-Ser-1490-LRP6 (relative to total LRP6) were also assessed to accurately determine that canonical Wnt signaling is active in the chick lung. Wnt stimulation leads to LRP6 phosphorylation at multiple sites (including Ser 1490) in its cytoplasmic region [6], which leads to the recruitment of Axin to the membrane, attenuation of β-catenin phosphorylation and consequently pathway activation.

**Figure 5:**
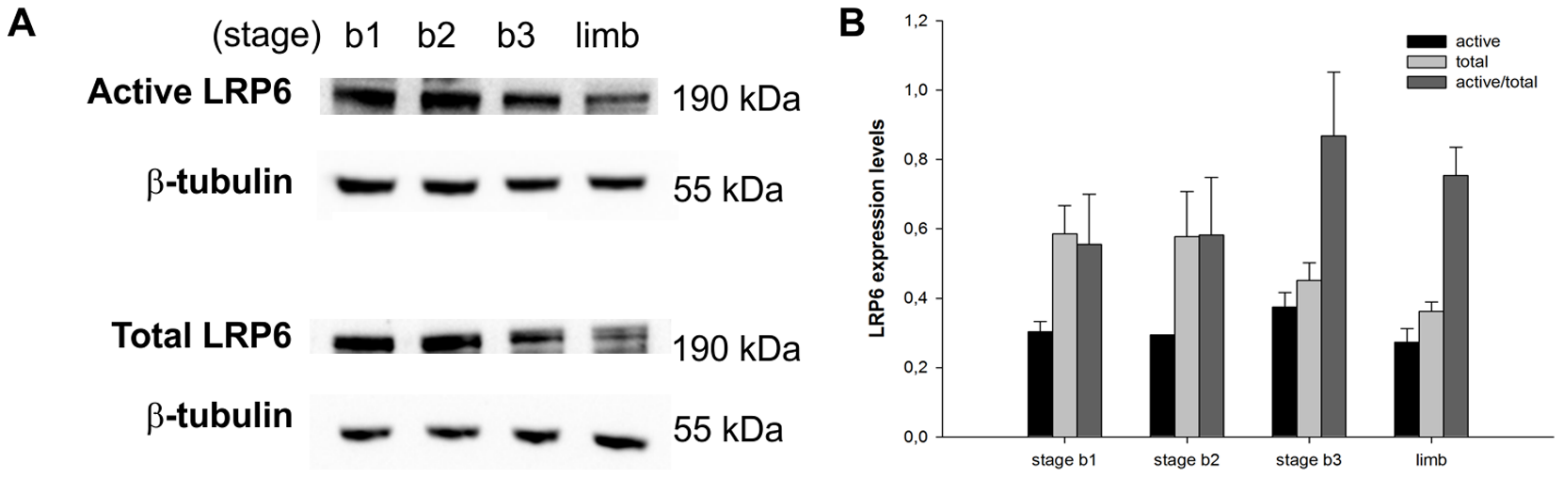
Activity of Wnt/β-catenin pathway in the embryonic chick lung. (A) Western blot analysis of phospho-LRP6 (Ser1490) and total LRP6 in stage b1, b2 and b3 lungs, and stage 24 limb (as positive control). Control loading was performed using β-tubulin (55 kDa). Phospho-Ser1490 and total LRP6 correspond to 190 kDa. (B) Semi-quantitative analysis for phospho-Ser1490 and total LRP6. Results are presented as arbitrary units normalized for β-tubulin.

Pooled samples of embryonic lungs (stage b1, b2 and b3) were used to assess protein expression levels of phosphorylated (active) and total LRP6 (Figure 5). As a positive control, pooled samples of embryonic chick limbs (HH24) were included. As it can be observed from Figure 5A, the active form of LRP6 is present in lungs from the three stages studied confirming that Wnt signaling is active in this embryonic tissue. Semi-quantitative analysis showed that there are no differences in active LRP6 expression levels between the three lung stages and limb; the ratio between active/total LRP6 suggests that the degree of pathway activation is the same in both tissues (Figure 5B).

### *In vitro* signaling inhibition

In order to assess the role of Wnt signaling pathway in early stages of chick lung development, *in vitro* inhibition studies were performed. *In vitro* inhibition of lung explants with FH535, a chemical inhibitor of the canonical Wnt signaling pathway, lead to an impairment of lung branching. The cell permeable sulfonamide FH535 affects β-catenin-mediated gene transcription by directly preventing the formation of β-catenin/TCF/LEF transcriptional complex. In fact, transcription of *TCF4* (a downstream target of canonical Wnt signaling) is suppressed in FH535-treated cells confirming Wnt signaling down-regulation [68]. Moreover, FH535 is potentially capable of attenuating transcription factor-mediated (i.e. TCF/LEF-dependent) nuclear translocation of β-catenin, contributing to Wnt signaling inhibition [69]. FH535 also targets PPAR (Peroxisome Proliferator-Activated Receptor, a member of the superfamily of nuclear receptors) signaling by preventing the recruitment of β-catenin to PPAR-γ and thus inhibiting β-catenin/PPAR-γ interaction [68]. A direct interaction between β-catenin and PPAR-γ has been described, suggesting a possible mechanism of cross-talk between the Wnt and the PPAR signaling pathways [70]. Depending upon the cell-type and system involved, both positive and negative interactions between PPAR-γ and Wnt signaling have been reported, and β-catenin seems to be the key Wnt signaling intermediate that mediates these interactions. For instance, it has been shown, in colon cancer cells, that β-catenin targets PPAR-γ activity by increasing PPAR-γ protein levels [70]. On the other hand, it has been described in embryonic human lung fibroblasts that PPAR-γ down-regulates β-catenin levels since it induces proteosomal degradation [71]. Taking this into account, inhibition of the Wnt/β-catenin pathway may involve modulation of the interaction between PPAR and β-catenin, however the molecular mechanism has not been investigated so far.

**Figure 6:**
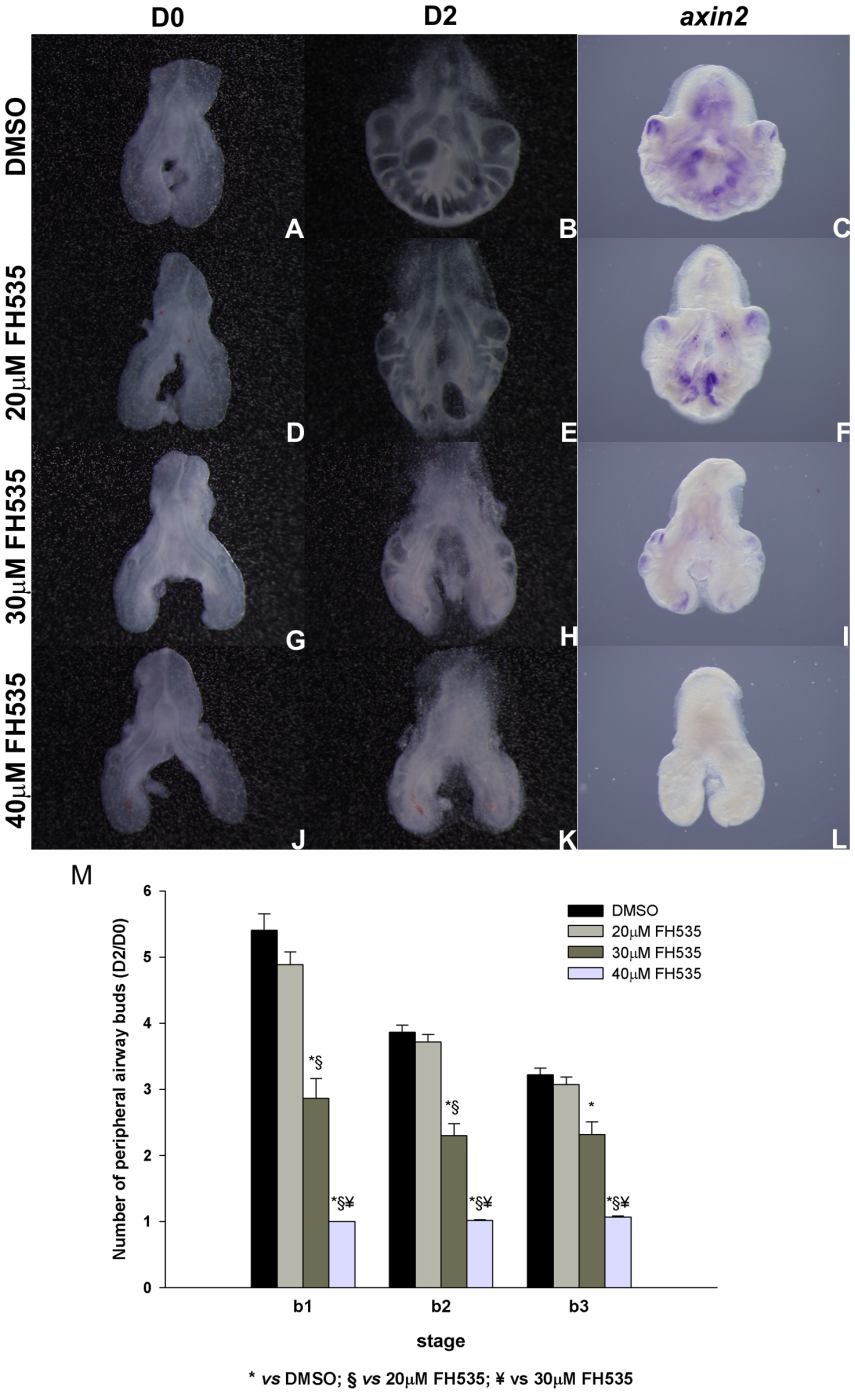
*In vitro* Wnt signaling inhibition (FH535) and branching analysis of lung explants. Representative examples of stage b2 lung explant culture, at D0∶0h (**A, D, G, J**) and D2∶48h (**B, E, H, K**) treated with DMSO (A, B), 20 µM (D, E), 30 µM (G, H) and 40 µM FH535 (J, K) and probed with *axin2* (**C, F, I, L**); n = 5 for each stage. Magnification: A, B, D, E, G, H, J, K –4x; C, F, I, L –5x. **M**: Branching analysis of stage b1 (n≥15 for each condition), b2 (n≥40 for each condition) and b3 (n≥30 for each condition) explants treated with DMSO and FH535 (20, 30 and 40 µM). Results are expressed as D2/D0 ratio. Data is represented as mean ± SEM. p<0.001 * *vs* DMSO, § *vs* 20 µM of FH535, ¥ *vs* 30 µM of FH535.

FH535 treated lungs present a decreased number of secondary buds when compared with controls (DMSO) (Figure 6). Branching analysis was performed and the results obtained are summarized in Figure 6M. Increasing doses of the inhibitor lead to a progressive decrease in the number of peripheral airway buds, as indicated by the D2/D0 ratio.

To demonstrate that this morphological alteration was due to Wnt signaling inhibition, b1 to b3 treated explants were probed with *axin2*, a direct readout of the canonical signaling pathway. In DMSO treated explants *axin2* is present and displays an expression pattern that is in accordance with the results described in the previous section (Figure 3U–X). On the other hand, FH535 treated explants present a reduction in *axin2* expression with increasing doses of inhibitor (Figure 6F, I, L). These results were consistent, independently of the stage. When compared to DMSO, 20 µM FH535 treated explants exhibit only a slight decrease in *axin2* expression level (Figure 6F) which indicates that Wnt signaling is not fully repressed in this condition also explaining the observed phenotype. On its turn, explants treated with 30 µM FH535 present a clear reduction of *axin2* expression level when compared with control explants (Figure 6I and 6C, respectively), indicating that Wnt signaling pathway is down-regulated in this condition which may account for the decrease in branching. When compared to DMSO, 40 µM FH535 treated explants lack *axin*2 mRNA (Figure 6L) supporting that Wnt signaling is clearly abolished in this condition.

**Figure 7:**
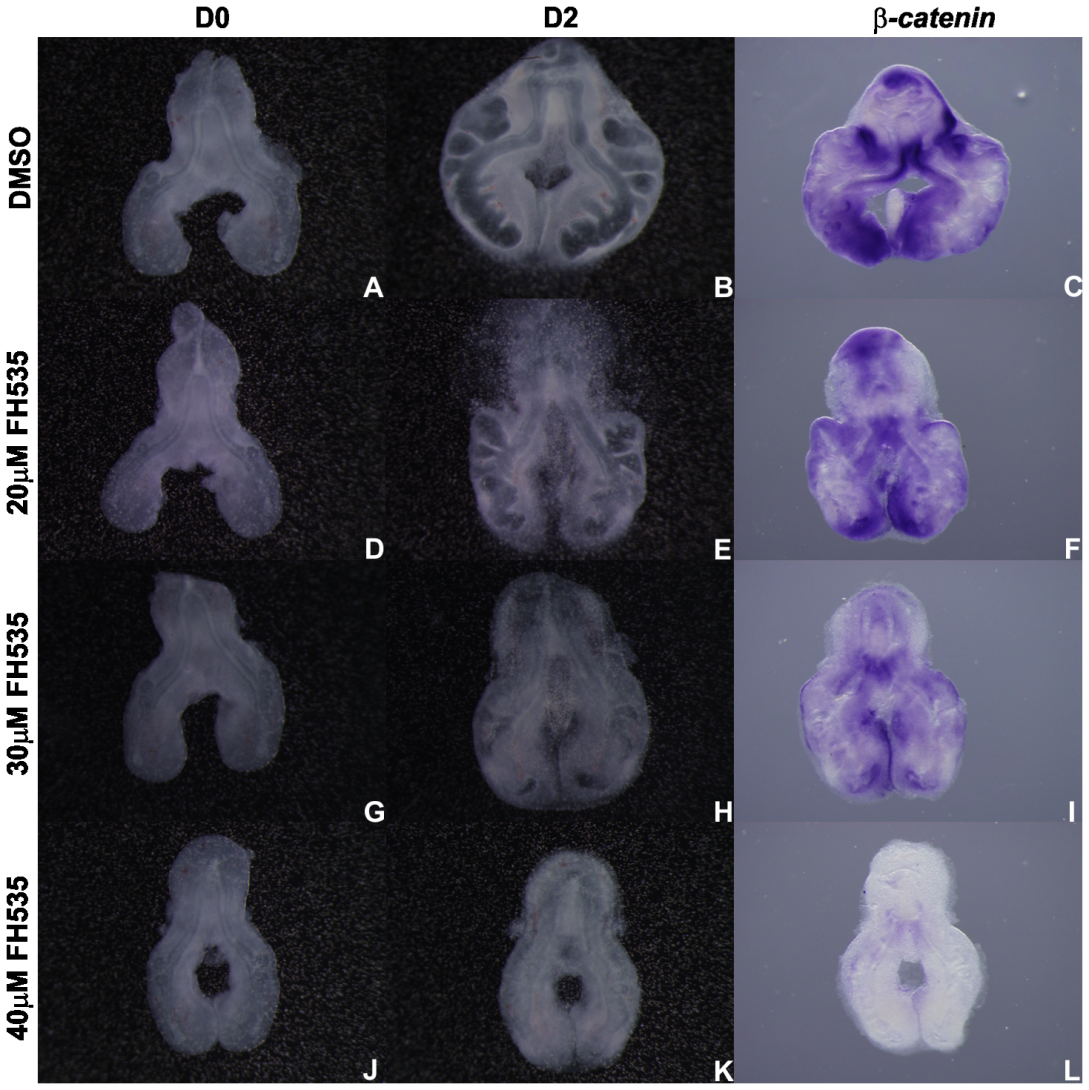
*In vitro* Wnt signaling inhibition (FH535) and *β-catenin* expression. Representative examples of stage b2 lung explant culture, at D0∶0h (A, D, G, J) and D2∶48h (B, E, H, K) treated with DMSO (A, B), 20 µM (D, E), 30 µM (G, H) and 40 µM FH535 (J, K) and probed with *β-catenin* (C, F, I, L); n = 5 for each stage. Magnification: A, B, D, E, G, H, J, K –4x; C, F, I, L –5x.

**Figure 8:**
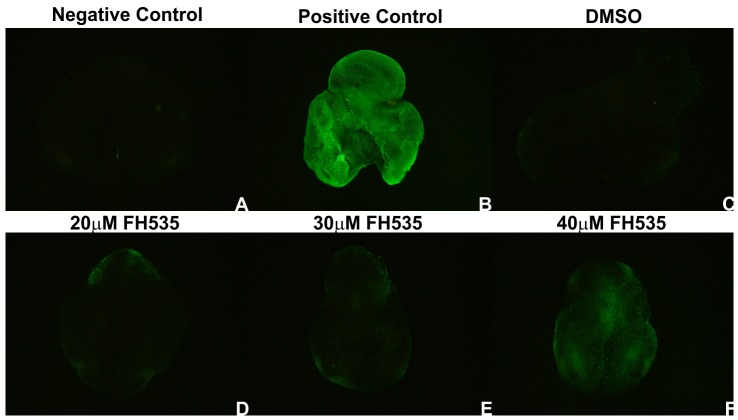
TUNEL assay in chick lung explants. Representative examples of negative (A) and positive control (B), DMSO (C), 20 µM (D), 30 µM (E) and 40 µM (F) FH535 treated stage b2 explants; n = 4 per condition. Negative control explants were incubated without TUNEL mix solution. Positive control explants were incubated with DNase at 37°C for 90 minutes.

In order to determine if FH535 supplementation lead to an unspecific decrease in gene expression, lung explants were probed with *β-catenin* (Figure 7). Explants treated with DMSO, 20 µM and 30 µM FH535 express *β-catenin* similarly. Only in the highest dose tested *β-catenin* expression is abolished. Since *β-catenin* is not a downstream target of Wnt signaling pathway, a complete abrogation of its expression was not expected. Therefore, with the purpose of establishing if this observation was due to cell death, a whole mount TUNEL assay in treated explants was performed (Figure 8). We found that apoptosis is absent in explants treated with DMSO (Figure 8C). Only minor apoptosis levels are observed in 20 µM and 30 µM FH535 treated explants in the most peripheral regions of the lung (Figure 8D and E, respectively). Regarding the 40 µM FH535 treated explants some degree of cell death is detected (Figure 8F). This observation might explain the absence of *β-catenin* expression in this experimental condition since this gene is not a direct downstream target of the Wnt signaling pathway, and point to a possible cytotoxic effect of FH535 in the highest dose tested.

The branching analysis, assessed by the number of new secondary formed after 48 hours in culture (D2/D0 ratio), confirmed that Wnt signaling inhibition impairs lung branching (Figure 6M). Lung explants treated with 20 µM of FH535 are similar to control (DMSO) explants and no significant statistical differences were found between them. On the other hand, 30 µM FH535 treated explants display a clear reduction in the number of secondary buds formed after 48 hours in culture. In the three stages studied, this dose induced a statistically significant decrease (p<0.001) when compared to control explants. These results are consistent with those published by De Langhe *et al.* [52], who described a decrease in lung branching in DKK1 treated explants. Dickkopf-1 (DKK1) is a potent and specific inhibitor of Wnt action that is secreted by the distal lung epithelium. DKK1 treated explants are characterized by a defect in cleft formation due to a decrease in fibronectin (FN) deposition [52]. The extracellular matrix protein FN secreted by lung epithelium is known to be a Wnt target gene in *Xenopus* [72], and is recognized as essential for cleft formation during the initiation of epithelial branching in several organ systems including the lung [73].

On the other hand, in the highest dose tested (40 µM FH535) no new secondary buds were formed, in the three stages studied. Nevertheless, this phenotype is most likely a result of not only Wnt inhibition but also to FH535 cytotoxic effect (Figure 8).

**Figure 9:**
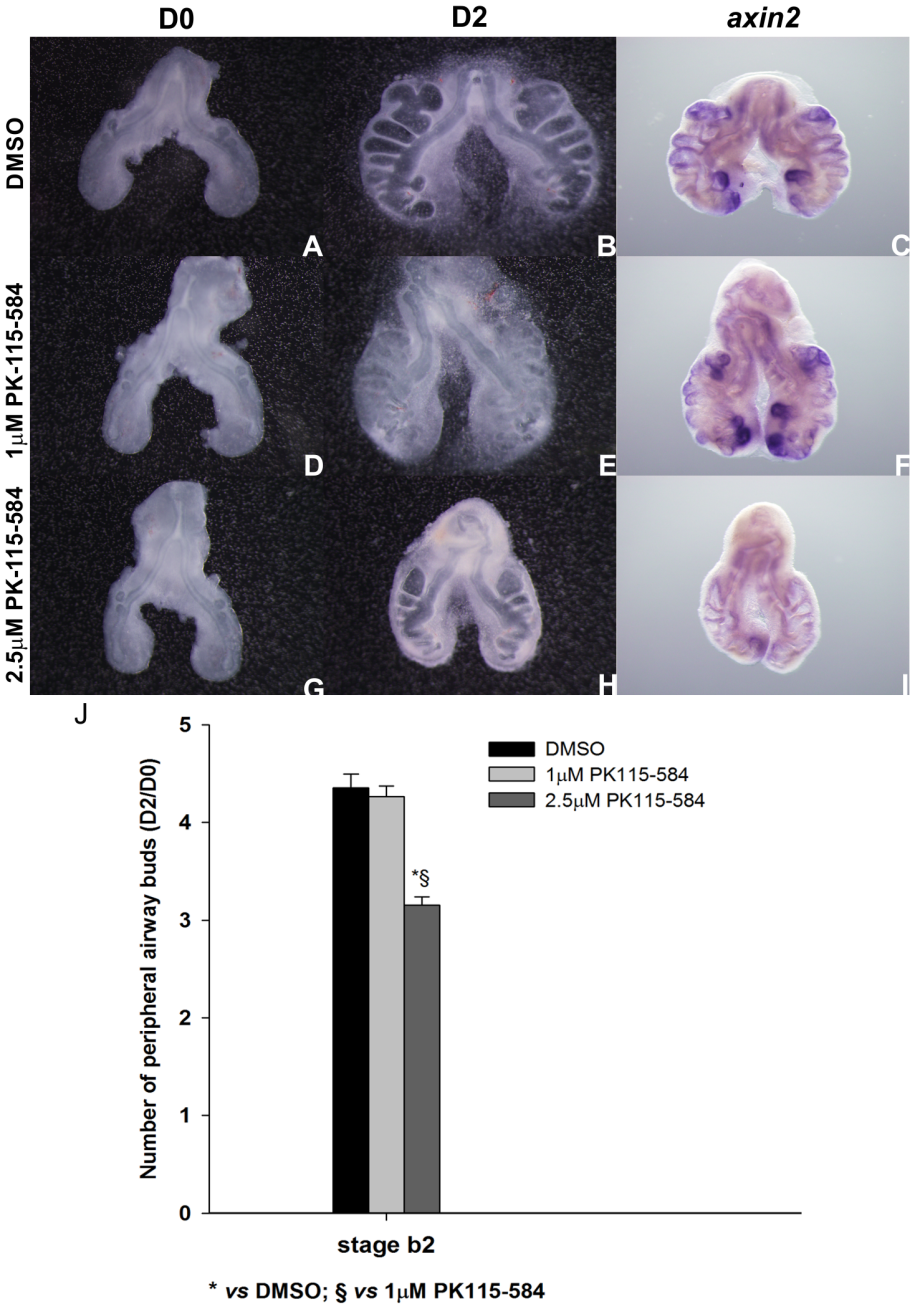
*In vitro* Wnt signaling inhibition (PK115-584) and branching analysis of lung explants. Representative examples of stage b2 lung explant culture, at D0∶0h (**A, D, G,**) and D2∶48h (**B, E, H**) treated with DMSO (A, B), 1 µM (D, E) and 2.5 µM (G, H) and probed with *axin2* (**C, F, I**); n = 4. Magnification: A, B, D, E, G, H –4x; C, F, I –5x. **M**: Branching analysis of stage b2 (n≥14 for each condition) explants treated with DMSO and PK115-584 (1 and 2.5 µM). Results are expressed as D2/D0 ratio. Data is represented as mean ± SEM. p<0.001: * *vs* DMSO, § *vs* 1 µM of PK115-584.

**Figure 10:**
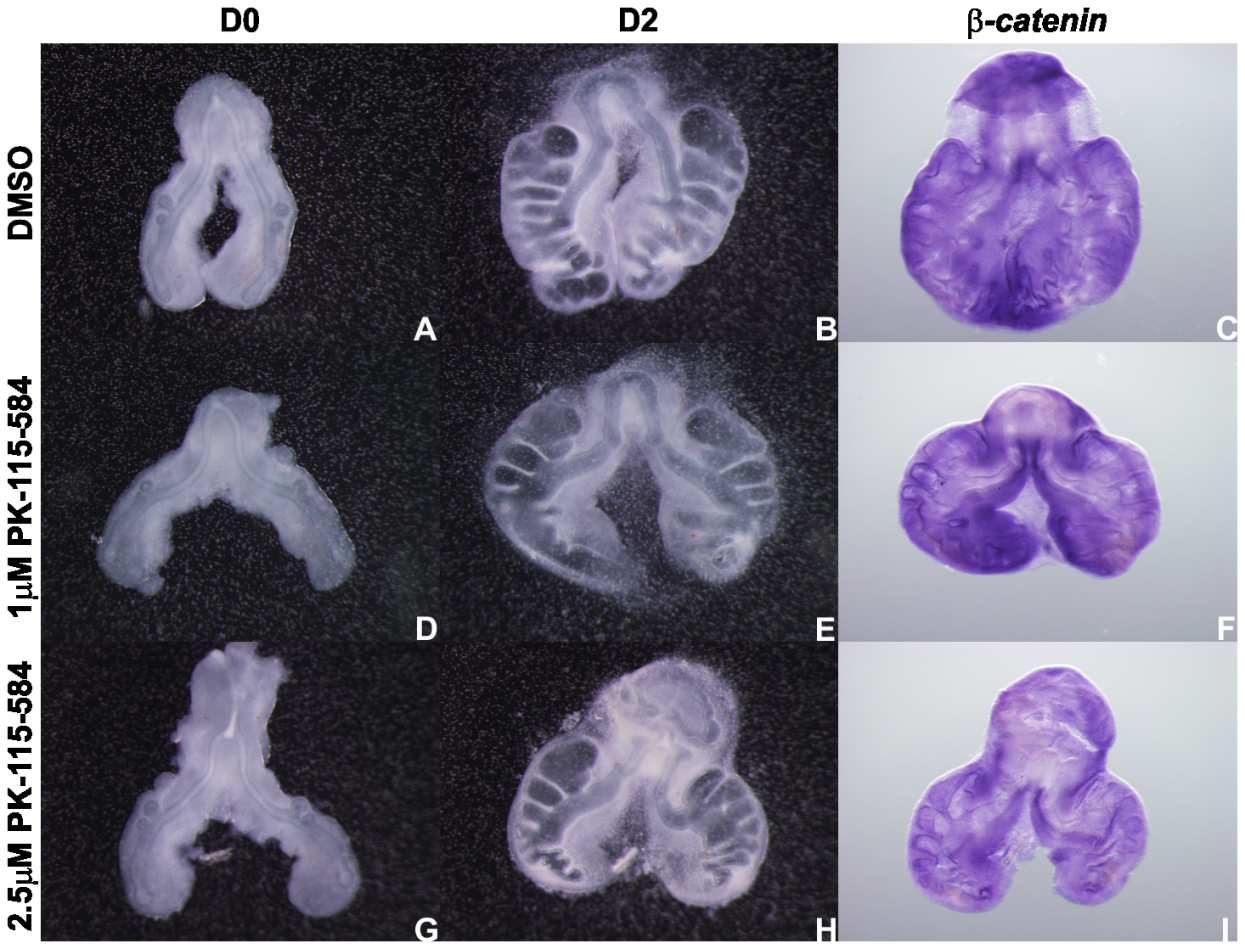
*In vitro* Wnt signaling inhibition (PK115-584) and *β-catenin* expression. Representative examples of stage b2 lung explant culture, at D0∶0h (A, D, G) and D2∶48h (B, E, H) treated with DMSO (A, B), 1 µM (D, E) and 2.5 µM (G, H) and probed with *β-catenin* (C, F, I); n = 4 for each stage. Magnification: A, B, D, E, G, H –4x; C, F, I –5x.

Taking into account the fact that FH535 is not absolutely specific for β-catenin/TCF4 interaction, and in order to corroborate the results obtained previously, b2 lung explants were treated with PK115-584. Since the previous results were consistent independently of the stage, only b2 lungs were assessed. PK115-584 prevents the association between Tcf4 and β-catenin and the expression of target genes [74]. This chemical inhibitor also targets protein kinase C (PKC) that is responsible for the inactivation of GSK3-β (the enzyme that targets β-catenin for degradation) [75]. These combined effects may explain the high potency of this compound in some assays. Lung explants treated with the highest dose tested of PK115-584 (2.5 µM) present a decreased number of secondary buds when compared with controls (DMSO) (Figure 9). Branching analysis was performed and the results obtained, D2/D0 ratio, are summarized in Figure 9J. Lung explants were probed with *axin2* to confirm Wnt signaling inhibition (Figure 9C, F and I) and with *β-catenin* (Figure 10C, F and I) in order to show that the inhibition is Wnt specific (since β-catenin is not a downstream target of this pathway). *axin2* expression levels are clearly reduced in 2.5 µM PK115-584 treated explants when compared with controls which confirms that Wnt signaling is inhibited; on its turn, *β-catenin* expression levels remain unaltered which demonstrated that this inhibition is Wnt specific. Therefore, these results validate the previous findings with FH535 and we can conclude that, also in the chick embryonic lung, Wnt signaling pathway in crucial for lung branching morphogenesis.

### Wnt-FGF crosstalk

**Figure 11:**
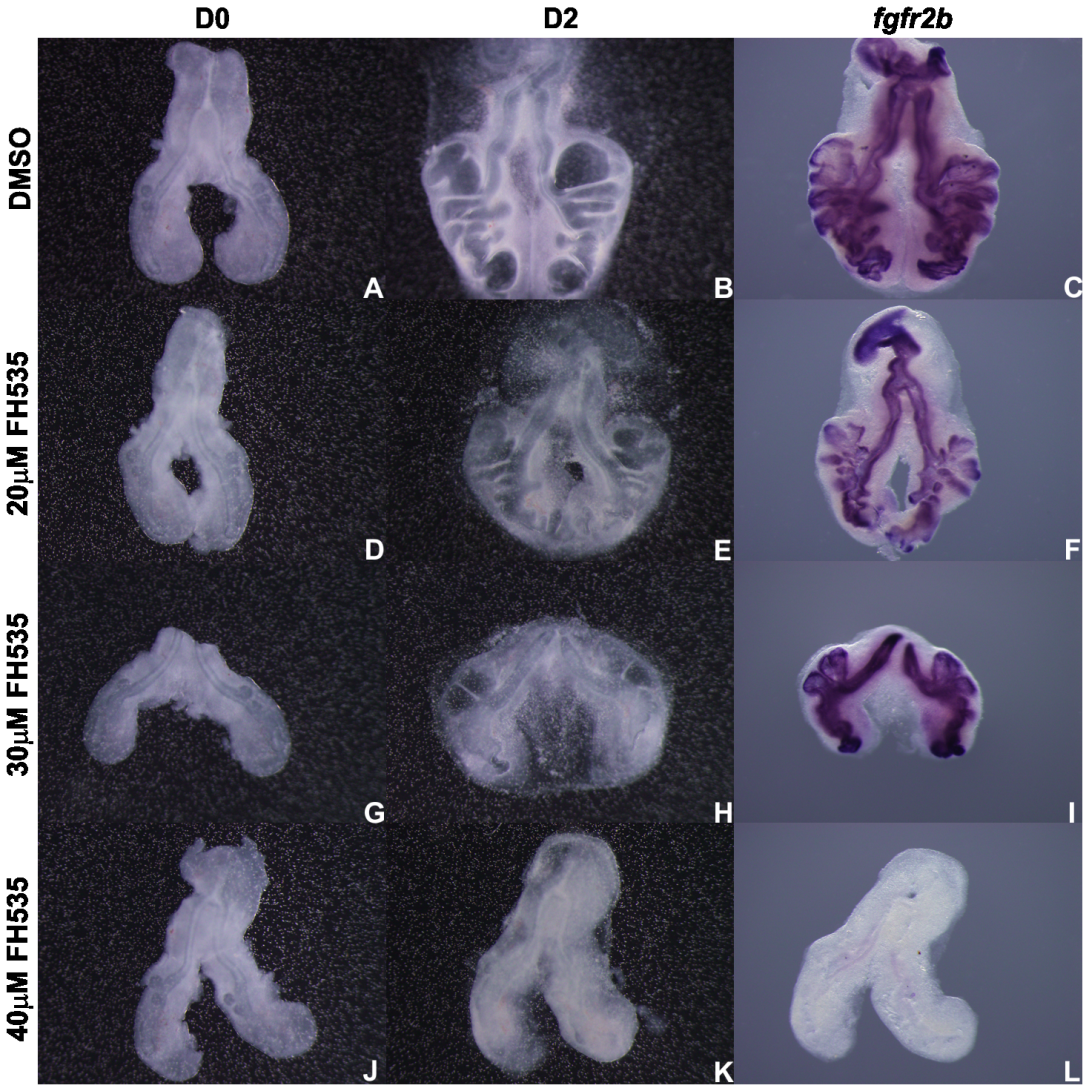
*In vitro* Wnt signaling inhibition and *fgfr2b* expression. Representative examples of stage b2 lung explant culture, at D0∶0h (A, D, G, J) and D2∶48h (B, E, H, K) treated with DMSO (A, B), 20 µM (D, E), 30 µM (G, H) and 40 µM FH535 (J, K) and probed with *fgfr2b* (C, F, I, L); n = 4 for each stage. Magnification: A, B, D, E, G, H, J, K –4x; C, F, I, L –5x.

**Figure 12:**
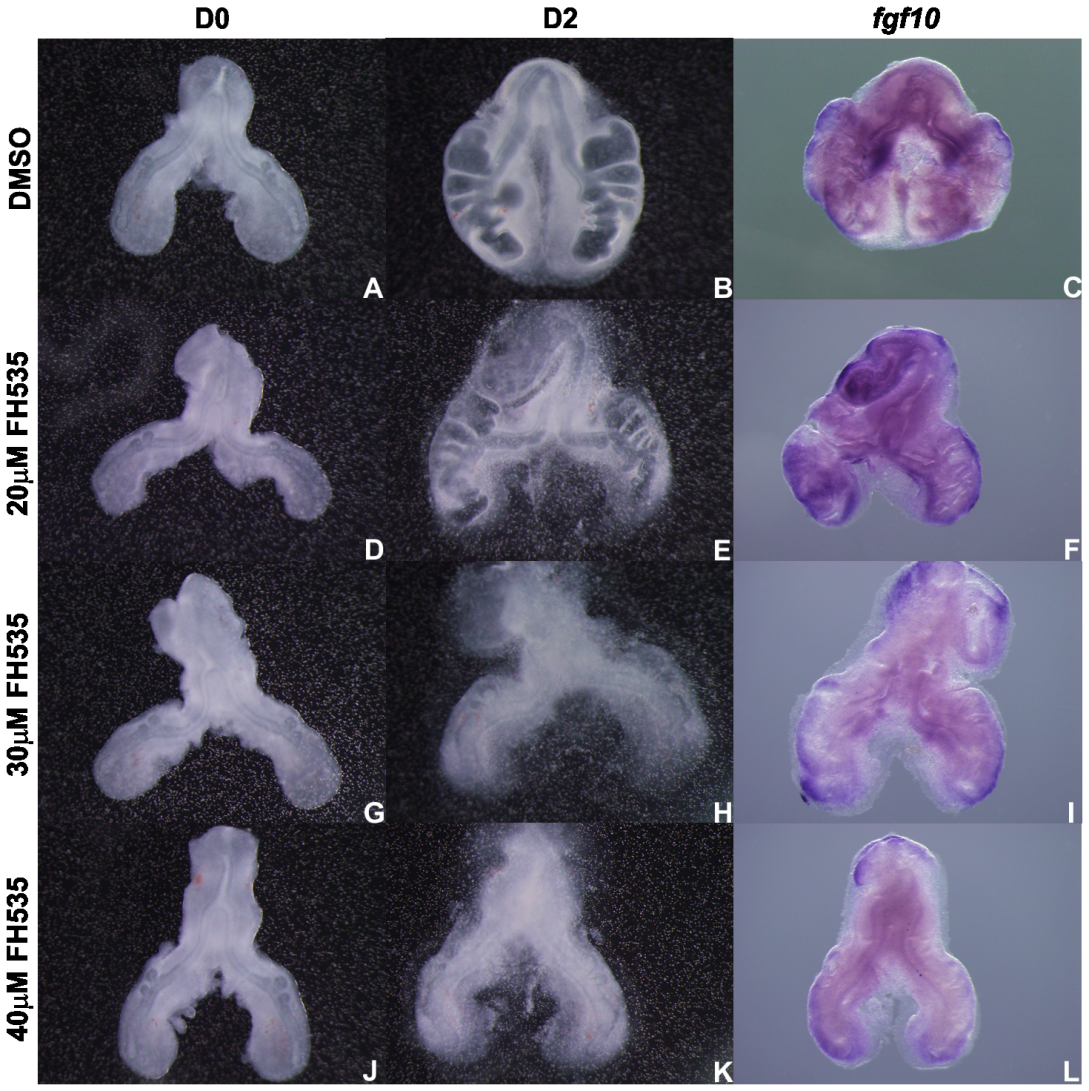
*In vitro* Wnt signaling inhibition and *fgf10* expression. Representative examples of stage b2 lung explant culture, at D0∶0h (A, D, G, J) and D2∶48h (B, E, H, K) treated with DMSO (A, B), 20 µM (D, E), 30 µM (G, H) and 40 µM FH535 (J, K) and probed with *fgf10* (C, F, I, L); n = 3 for each stage. Magnification: A, B, D, E, G, H, J, K –4x; C, F, I, L –5x.

**Figure 13:**
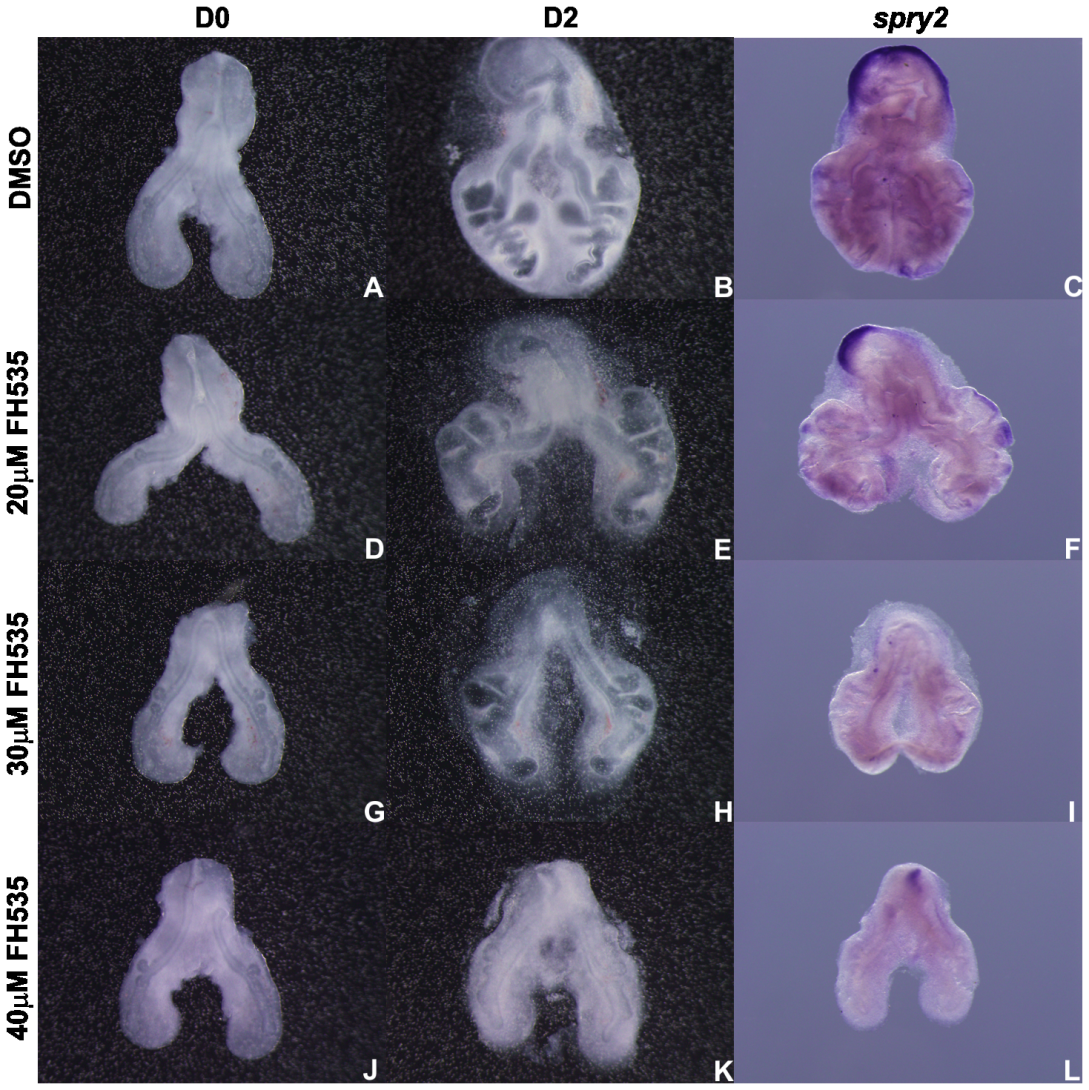
*In vitro* Wnt signaling inhibition and *spry2* expression. Representative examples of stage b2 lung explant culture, at D0∶0h (A, D, G, J) and D2∶48h (B, E, H, K) treated with DMSO (A, B), 20 µM (D, E), 30 µM (G, H) and 40 µM FH535 (J, K) and probed with *spry2* (C, F, I, L); n = 3 for each stage. Magnification: A, B, D, E, G, H, J, K –4x; C, F, I, L –5x. The signal observed in the most proximal region of the lung is due to the accumulation of developing solution.

Recent literature suggested a link between canonical Wnt signaling and FGF signaling [24], [76]. For this reason, Wnt-FGF crosstalk was assessed in lung explants. For this purpose, FH535 treated lung explants were probed with *fgfr2b*, *fgf10* and *spry2* (Figure 11 to 13, respectively). It seems clear that *fgfr2b* expression levels remain virtually unchanged in lung epithelium, in explants treated with 20 µM and 30 µM FH535 (Figure 11F and I, respectively). This fact differs from the one described by Shu *et al.* that demonstrated, by *in situ* hybridization, that *fgfr2* expression is reduced in lung airway epithelium in the absence of Wnt/β-catenin signaling [24]. Regarding *fgf10*, expression seems slightly diminished in the dorsal mesenchyme adjacent to the emerging secondary buds, in 30 µM FH535 treated explants when compared with DMSO explants (Figure 12I and C, respectively). A study from De Langhe and co-workers has shown that canonical Wnt signaling inhibition in murine lung explants treated with DKK1 (an inhibitor of Wnt signaling pathway) resulted in branching impairment but *fgf10* expression was retained [52]. Concerning *spry2* expression, a clear reduction in its expression, in distal epithelium and the epithelium of the emerging secondary buds, is observed in 30 µM FH535 treated explants (Figure 13I).

The inhibition of Wnt signaling in chick and mouse lung leads to a decrease of the downstream targets of FGF signaling (*spry2* levels and phospho-ERK1/2 activity, respectively). Our data indicates that Wnt-FGF crosstalk is conserved and that, also in the chick lung, Wnt signaling acts upstream FGF signaling.

In light of these results we consider that the observed decrease in lung branching is a Wnt dependent response and not primarily a consequence of decreased FGF signaling. To corroborate this hypothesis is the fact that lung explants treated with FH535 or PK115-584 display a decrease in branching but do not exhibit a cystic shape. Conversely, *in vitro* inhibition of FGF signaling by a FGF receptor antagonist leads to abnormal/cystic secondary buds [27]. If FGF signaling was the major contributor in these experimental conditions, lung explants would most likely be similar to the previously described phenotype.

The architecture of the bronchial system in the avian lung fundamentally differs from the mammalian lung. Unlike murine lung where the airway system forms by dichotomous bifurcation [30], in the avian lung, a continuous bronchial system forms by sprouting of the secondary bronchi from the primary bronchus [77]. After that, the parabronchi interconnect the secondary bronchi, establishing continuity of the bronchial system [77]. While the respiratory tree of the mammalian lung is blind-ended (alveoli), in the avian lung the air capillaries are essentially continuous anastomosing air conduits. Despite these differences, chick lung development seems to follow a pattern somewhat similar to mammalians. The understanding of the specifics of chick lung development will allow us to better comprehend the mechanisms responsible for divergence between mammals and birds. On the other hand, the processes shared by both models, will most likely represent crucial regulatory mechanisms in normal development.

In conclusion, our work demonstrates the importance of Wnt signaling in the epithelial-mesenchymal interactions that determine epithelial branching and mesenchyme growth and consolidate our understanding of the activity of Wnt signaling pathway in early chick lung branching.
